# Transitional care programs: who is left behind? A systematic review

**DOI:** 10.5334/ijic.805

**Published:** 2012-08-10

**Authors:** Emily Piraino, George Heckman, Christine Glenny, Paul Stolee

**Affiliations:** School of Public Health and Health Systems, University of Waterloo, Waterloo, Ontario, Canada; School of Public Health and Health Systems, Schlegel – University of Waterloo Research Institute for Aging, University of Waterloo, Waterloo, Ontario, Canada; Faculty of Medicine, McMaster University, Hamilton, Ontario, Canada; School of Public Health and Health Systems, University of Waterloo, Waterloo, Ontario, Canada; School of Public Health and Health Systems, University of Waterloo, Waterloo, Ontario, Canada

**Keywords:** systematic review, care transitions, self-care, comorbidity, rehospitalization

## Abstract

**Objective:**

Older adults are at risk of rehospitalization if their care transitions from hospital-to-home are not properly managed. The objective of this review was to determine if older patient populations recruited for randomized controlled trials of transitional care interventions represented those at greatest risk of rehospitalization following discharge. Relevant risk factors examined were cognitive impairment, depression, polypharmacy, comorbidity, length of stay, advanced non-malignant diseases, and available social support.

**Design:**

Systematic review.

**Setting:**

Hospital to home.

**Participants:**

Older hospitalized adults.

**Measurements:**

For inclusion, articles were required to focus on hospital-to-home transitions with a self-care component, have components occurring both before and after discharge, and a randomized controlled trial design. Articles were excluded if participants had a mean age under 55 years, or if interventions focused on developmental disabilities, youth, addictions, or case management, or were solely primary-care based.

**Results:**

Following title, abstract, and full review by two authors, 17 articles met inclusion criteria. Risk factors for rehospitalization were often listed either as exclusion criteria or were not reported at baseline by the studies. One study included patients with all identified risk factors for rehospitalization.

**Conclusions:**

These data suggest that published studies of transitional care interventions do not often include older adults at highest risk of rehospitalization, raising concerns about the generalizability of their results. Studies are needed that evaluate interventions that explicitly address the needs and characteristics of these patients.

## Introduction

With population aging, many countries are facing increased pressures on health care resources. Older adults with chronic disease are the fastest growing segment of the population and the heaviest health care users, accounting for up to four times more hospital days than the rest of the population [1]. In order to address hospital bed overflow, hospitals are discharging patients earlier with the expectation that a portion of their care will take place in the next care setting. For older patients with multiple chronic conditions that require close follow-up, early discharge may be associated with an increased risk of rehospitalization.

Care transitions across different health care settings may be particularly problematic for vulnerable older adults, who may be at risk for poor health outcomes or worsening of their conditions [2–8]. Care fragmentation leaves patients and their caregivers unprepared to manage their conditions following hospital discharge [3, 9], leading to greater use of hospital, emergency, post-acute and ambulatory services [8, 10]. Nearly one-quarter of older patients discharged from the hospital experience an adverse health outcome such as rehospitalization within 30 days [11]. Rehospitalizations place older adults at risk for further health declines that threaten functional independence and quality of life, and that may lead to unnecessary hospital bed use, premature institutionalization, and costs to the health care system [11–13].

Transitional care interventions are designed to address the need for better care coordination, recognizing that patients and their caregivers are the common factors across care settings and therefore key players in care transition management [3]. Hospital-to-home transitions require that patients acquire self-care skills for conditions that are either newly diagnosed or that have recently worsened [10, 14]. Self-care aims to improve health outcomes and prevent unnecessary hospitalization [15] through symptom management and promotion of lifestyle, physical and psychosocial changes that are necessary to manage their conditions [14]. Self-care skills are essential for community-dwelling patients who may not have access to ongoing nursing support. Current literature suggests that transitional care interventions based on self-care skills may be an effective solution for improving patient and system outcomes [2, 5, 16–19].

Risk factors for rehospitalization include older age, inadequate support systems, multiple comorbidities, polypharmacy, depression, functional impairment, low self-health rating, and history of non-adherence [3, 20–28]. For transitional care interventions to be most effective, they must target patients at risk of unplanned rehospitalization. An article recently published by Naylor [20] suggests that discharge planning and follow-up procedures used in some transitional care interventions focus on patients with single conditions, and are thus not targeting older patients with complex multiple conditions. Care interventions need to address the complex needs of at-risk older adult patients while ensuring continuity of care in a diverse range of settings and across providers [20], rather than treating patient conditions in isolation. The objective of this review is to determine if patients included in studies of transitional care interventions are truly at high risk for rehospitalization.

## Methods

All relevant English language articles published up to and including August 2011 were considered for this review. Criteria to establish article relevance are defined below.

Inclusion criteria:

1. Randomized controlled trials of transitional care interventions for patients moving from hospital to home.
2. Self-care was an integral component of interventions studied [8, 15].
3. Interventions included components occurring both before and after hospital discharge.
4. Trials assessed the efficacy of interventions on patient and/or system outcomes (e.g. rehospitalizations, emergency department visits, mortality, home visits, costs, care transition quality, satisfaction, mortality, quality of life, falls, adherence to treatment, service use, and cost).

Exclusion criteria:

1. Study populations had a mean age under 55 years [29].
2. Articles describing transitional care interventions designed exclusively for developmental disabilities, youth and/or addictions.
3. Articles describing programs that focused exclusively on providing patient and/or system management (e.g. case management interventions).
4. Articles describing programs based solely in primary care [30].

For the purpose of this review, transitional care interventions were defined as a structured set of services to enhance the health, safety, and continuity of care for patients moving from hospital to home [20, 23]. We chose to focus on community-dwelling seniors, rather than long-term care residents, as they make up the majority of older adults discharged from hospital and for whom the acquisition of self-care skills is a reasonable expectation [22, 24]. Self-care was defined as “enhancing the ability of patients and informal caregivers to manage chronic illness, including learning to recognize and manage disease exacerbations and access the system early enough to avert acute care use” [15]. Although discharge planning is a vital part of successful care transitions, we will focus on models with components before and after hospital discharge [2].

Articles were retrieved from Medical Literature Analysis and Retrieval System Online (Medline), Cumulative Index to Nursing and Allied Health Literature (CINAHL), and Embase for all available years up to and including August 2011. Search strings were tested using previously identified relevant articles and are included in Appendix A. Article titles and abstracts were reviewed for relevance based on the inclusion and exclusion criteria listed above. Relevant articles were independently reviewed by two authors, with a third reviewer adjudicating in the case of disagreement. For each article selected, information was gathered and summarized in two tables. A list of risk factors that should be considered when assessing the real-world generalizability of transitional care interventions was developed based on a review of the literature [20–28]. This list was used to gauge whether articles reviewed targeted patients with risk factors for rehospitalization following hospital discharge, including:

1. Comorbidity: occurrence of two or more medically diagnosed conditions [22, 24];
2. Polypharmacy: concurrent use of two or more drugs [25];
3. Cognitive impairment of any severity [24, 26, 28];
4. Depression [27, 28];
5. Inadequate social support [20, 21]; and
6. Patients with advanced non-malignant diseases [31, 32].

Information was abstracted from each of the reviewed articles including study population characteristics, components of the interventions, outcome measures, and risk factors for rehospitalization, including risk factors that were: 1) directly specified; or 2) not specified but present among participants included in each study.

## Results

### Summary of search results

**Figure 1.**
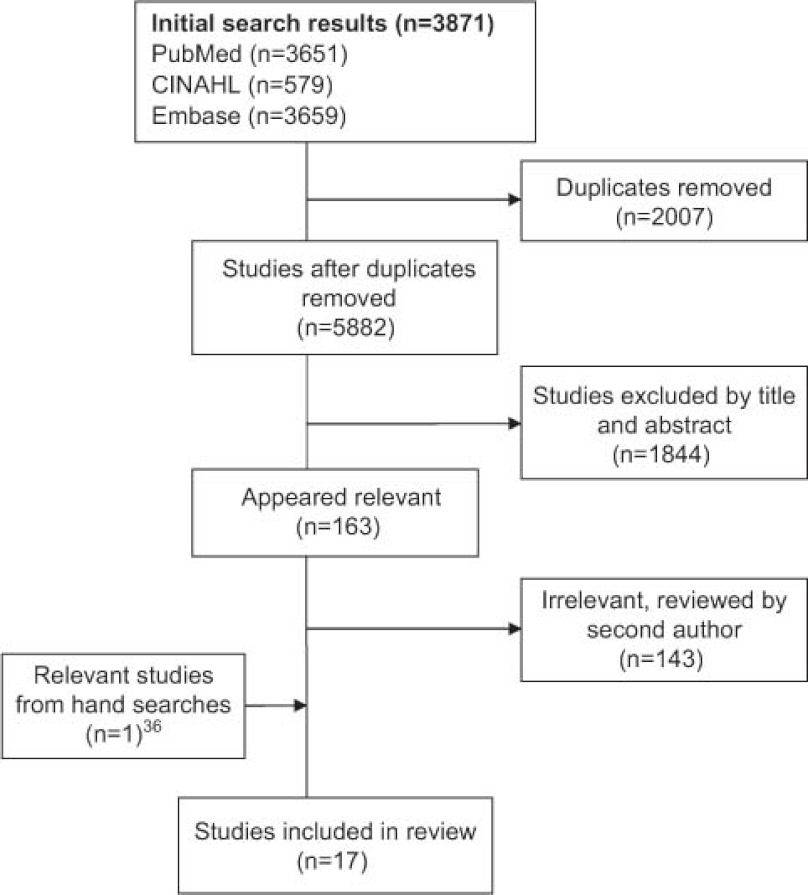
Results of Search Strategy. This figure describes how articles were chosen from our initial search.

After removing duplicates, the initial key word search identified 5882 articles (Figure 1). Based on the above criteria, 163 studies were identified as relevant based on title and abstract content and were reviewed by two authors (EP and CG), yielding a final list of 17 articles.

Summary information for each article including participant selection criteria, baseline characteristics, and components of each transitional care intervention can be found in Appendix B. Six studies were completed in the US, four in Europe (Spain, Ireland, Netherlands, and UK), two in Canada, and two in Asia (Taiwan and Hong Kong). Nine articles specifically targeted older patients [2, 6, 18, 33, 34], ten articles focused on patients with heart failure, and one article focused on patients with hip fracture. The mean age of the participants in the intervention group range from 69 years to 79 years across studies. For a summary of risk factors targeted by each intervention, see Table 1. Table 2 summarizes the characteristics of the study population by article.

### Cognitive impairment and depression

One [35] of the fifteen studies explicitly reported on cognitive status and depression in their sample at baseline. Eight [2, 6, 17, 18, 36–38] studies excluded patients with cognitive impairment and three [16, 34, 39] excluded those with dementia. Fourteen studies did not provide data on depression and three did not report on cognitive impairment or dementia [18, 33, 40].

### Comorbidities

Three articles [2, 5, 35] specifically targeted participants with two or more comorbid conditions. Two studies [17, 35] provided information on the number of comorbid conditions in the sample at baseline; the remaining articles either did not provide data on the comorbid status of their participants or did not include participants with more than one diagnosed condition.

### Polypharmacy

One study [35] specifically targeted patients taking two or more medications; three of the remaining articles [2, 5, 17] reported the mean number of medications taken by the participants at baseline. Four of the articles did not provide [6, 18, 33, 34] any data on number of medications taken by their study population and the remaining articles reported on the proportion of the study population currently prescribed specific types of medications (usually those related to heart failure and other cardiac conditions) [16, 19, 36, 38, 39].

### Social support

Nine studies either specifically targeted or included patients living alone or those with little or no social support [2, 5, 6, 19, 35, 36, 38, 40, 41]. Conversely, 96.8% of the sample used by Huang and Liang [18] lived with family, and in the study by Zhao and Wong [34], one % of participants lived alone.

### Advanced non-malignant disease

Ten studies excluded patients requiring hospice-palliative care or with a life-expectancy estimated at <3 to 6 months who had non-malignant diseases [6, 16–19, 35–39].

In summary, four [2, 5, 17, 35] of the studies reviewed targeted and/or included study participants with three or more risk factors for rehospitalization, while the remaining ten explicitly included at most one risk factor. Three interventions [2, 6, 35] included patients with all risk factors though only one [35] study *a priori* defined these criteria explicitly.

## Discussion

Transitional care interventions aim to support patients being discharged from hospital back to the community by focusing on enhancing self-care abilities among patients and caregivers, thus improving health outcomes and preventing unnecessary rehospitalizations. A recent systematic review of published randomized controlled trials suggests that transitional care programs can indeed achieve these goals [42]. We conducted a systematic review of randomized controlled trials of transitional care programs in order to determine how well patients enrolled in these trials correspond to patients known to be at high risk for rehospitalization. Our data suggest that significant differences exist between these patient populations, raising concerns about the generalizability of the interventions studied and their actual potential to improve outcomes among patients at highest risk of rehospitalization.

### Cognitive impairment and depression

An important gap relates to cognitive impairment and depression. These conditions are frequent in older adults and will become increasingly prevalent with population aging [43]. Dementia is a key factor associated with many negative patient and system level outcomes, including incontinence, falls, deconditioning, increased ‘alternate level of care’ days (acute care days after acute care is no longer needed), greater length of stay and rehospitalization [22, 44–46]. Health outcomes are even poorer for older patients with concomitant cognitive impairment and depression, particularly during care transitions [46, 47].

### Comorbidities

Similarly, few of the studies reviewed explicitly targeted older patients with multiple co-existing medical conditions, casting doubts about whether transitional care interventions benefit such patients. It is estimated that 65% of adults aged 65–79 and 78% of adults aged 80 and over have two or more comorbidities, with the largest subgroup in each age category consisting of those with four or more chronic conditions [48]. In fewer than half of the studies reviewed did patient characteristics reflect a relevant comorbidity distribution. Many studies that described the comorbid status of their participants only focused on major medical conditions such as hypertension, heart disease, and diabetes mellitus. In many older patients, geriatric syndromes, such as falls, incontinence, disability, weight loss, dizziness, vision and hearing problems, that frequently co-exist with chronic illnesses, were not reported [49]. Geriatric syndromes are associated with a degree of disability comparable to major medical illnesses, and may have a comparable impact on health outcomes [49].

### Polypharmacy

Adverse drug reactions and poor adherence are common concerns associated with polypharmacy, particularly among older patients with multiple comorbidities [50]. Adverse drug events following hospital discharge often reflect poor communication related to medications [51]. Though improved medication management was a frequently stated goal of transitional care studies reviewed, only one explicitly targeted participants taking more than one medication, while over one quarter provided no data on the number of medications prescribed to their study population.

### Social support

Patients with inadequate social support, such as living alone, being unmarried, having irregular family contact, and being home alone for more than two hours daily, were targeted by nine of the studies reviewed. Social support has been shown to be important in ensuring better medication and treatment adherence, and reducing the risk of hospitalization [52–54].

### Advanced non-malignant diseases

Patients described as palliative or ‘terminal’ (defined as death being imminent or a life expectancy less than one year) were excluded from ten of the studies reviewed. This may be problematic with respect to advanced non-malignant conditions such as heart failure, for which accurate prediction of life-expectancy at the individual level is difficult [55]. Patients with advanced heart failure are at particularly high risk of rehospitalization, and their potential exclusion from a transitional care intervention based on an inaccurate estimate of life expectancy may not be justifiable. Evidence from an RCT of a disease management program for palliative care patients with both malignant and advanced non-malignant diseases suggests that even among such patients, programs focused on enhancing self-care and symptom management can reduce the risk of rehospitalization [55]. The Canadian Cardiovascular Society Heart Failure Management Guidelines (2008) [56] recognizes that patients with advanced heart failure are likely to benefit from transitional care programs.

### Study limitations

This review has several limitations. Transitional care is an evolving concept with many definitions, approaches and levels of involvement. Our results are therefore not applicable to transitional care of patients in other age groups or between settings other than hospital and community. Restricting this review to randomized controlled trials may have excluded other transitional care studies evaluated using different methods, but which may have yielded informative results.

### Conclusion

In summary, most of the transitional care interventions described in this review did not explicitly focus on older patients with risk factors for rehospitalization, such as cognitive impairment, dementia, depression, multiple comorbidities, polypharmacy, or nearing the end-of-life. Therefore, the results of published transitional care interventions are not readily generalizable to patients at highest risk of rehospitalization. Transitional care interventions should be developed and evaluated for such high-risk populations, as emphasized by Ferruci et al. [55] who advocated the creation of guidelines for trials to target higher-risk older adults, such as those with dementia, and cautioned that overly restrictive inclusion criteria due threaten the generalizability of trials that fail to adhere to such guidelines.

## Acknowledgements

This research was supported by an Emerging Team Grant from the Canadian Institutes of Health Research. We are grateful to Jackie Stapleton for her assistance with the search strategy.

## Author contributions

All authors meet the criteria for authorship stated in the Uniform Requirements for Manuscripts Submitted to Biomedical Journals.

**Emily Piraino:** Conception and design, acquisition of data, interpretation of data, primary author of article content, approved final version submitted.

**Dr. George Heckman:** Conception and design, interpretation of data, revising article, and final approval of version submitted.

**Christine Glenny:** Conception and design, data analysis, drafting article, final approval of version submitted.

**Dr. Paul Stolee:** Conception and design, data interpretation, revising article, final approval of version submitted.

No sponsor was included.

## Reviewers

**Bianca Buurman,** RN, PhD, AMC, Department of internal medicine, section of geriatric medicine, P.O. Box 22660, 1100 DD Amsterdam, Netherlands.

**Sherry L. Grace**, PhD, Associate Professor, Faculty of Health, York University, Bethune 368, 4700 Keele Street, Toronto, ON M3J1P3.

**Ingrid Mur-Veeman**, Department of HSR, Maastricht University, School for Public Health and Primary Care (CAPHRI), Faculty of Health, Medicine and Life Sciences, P.O. Box 616, 6200 MD Maastricht, Netherlands.

## Figures and Tables

**Table 1.**
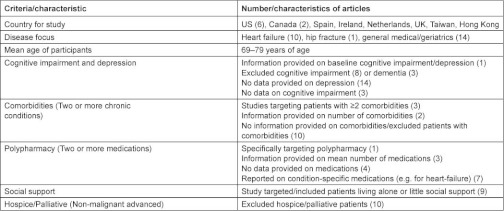
Summary of key study characteristics and targeted risk factors for hospitalization

**Table 2.**
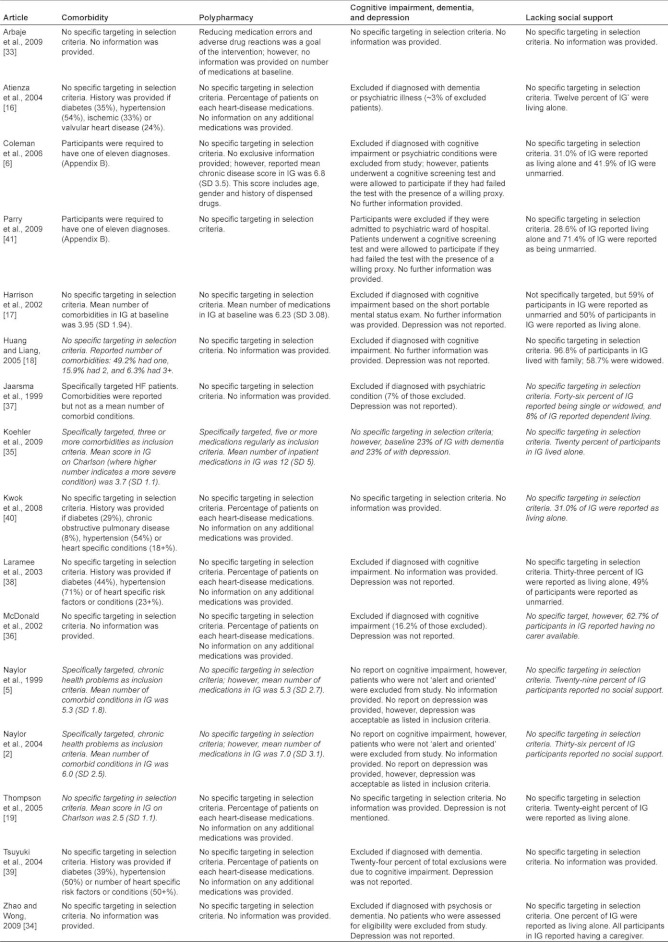
Summary of participants by study

Italicized font indicates that the intervention successfully targeted the group indicated based on our criteria.

## Appendix A Search string used in medline

### Search 1:

“Case management” [MESH] OR “case management” [TIAB] OR “case manager” [TIAB] OR “care management” [TIAB] OR “care manager” [TIAB] OR “patient discharge” [MESH] OR “transition” OR “transitions” OR “transitioning” OR “patient transfer” OR “hand off” OR “hand-off” OR “transitional” OR “follow-up care” OR “follow-up service” OR “discharge planning” OR “post-discharge support” OR “hospital discharge”.

### Search 2:

Hospital [TIAB] OR hospital [MESH] OR “medical centre” OR “clinic” OR “health service” OR “hospitalization” OR “acute care” OR “postacute”.

### Search 3:

Search 1 + Search 2 AND “randomized”.
